# Primary Cutaneous Follicle Center Lymphoma of the Nasal Dorsum and Tip Treated by Surgical Excision and Full-Thickness Skin Graft Reconstruction: A Case Report

**DOI:** 10.7759/cureus.101928

**Published:** 2026-01-20

**Authors:** Carina M Álvarez-Dávalos, Erick M Hernández-Mancillas, Gustavo García-Marín, Hector Alvarez-Trejo, Quitzia L Torres-Salazar

**Affiliations:** 1 Surgery, Centro Universitario de Ciencias de la Salud, de la Universidad de Guadalajara, Guadalajara, MEX; 2 Surgery, Universidad Autónoma de Durango, Durango, MEX; 3 Surgery, Instituto de Seguridad y Servicios Sociales de los Trabajadores del Estado, Guadalajara, MEX; 4 Biomedical Sciences, Universidad Juárez del Estado de Durango, Durango, MEX

**Keywords:** full-thickness skin graft, nasal lymphoma, primary cutaneous b-cell lymphoma, primary cutaneous follicle center lymphoma, primary cutaneous lymphoma, surgical excision

## Abstract

Primary cutaneous follicle center lymphoma (PCFCL) is an indolent subtype of primary cutaneous B-cell lymphoma that may present diagnostic challenges due to its variable clinical appearance and frequent overlap with benign inflammatory or neoplastic skin conditions. We report the case of a 79-year-old male patient who presented with a persistent pruritic and erythematous lesion involving the nasal dorsum and tip. Initial dermatologic evaluation and limited skin biopsy failed to establish a definitive diagnosis, and the lesion was managed conservatively without improvement. Given persistent symptoms and anatomical involvement, wide surgical excision with oncologic intent was performed after systemic staging excluded extracutaneous disease. Immediate reconstruction was achieved using a full-thickness skin graft harvested from an upper blepharoplasty performed in the same operative session. Histopathological examination of the excised specimen confirmed the diagnosis of PCFCL with tumor-free margins. Postoperative recovery was uneventful, and at 12-month follow-up, the patient demonstrated satisfactory functional and aesthetic outcomes with no evidence of local recurrence. This case highlights the potential for PCFCL to masquerade as a benign inflammatory condition, the limitations of small diagnostic biopsies, and the importance of adequate tissue sampling. It also underscores the role of surgical excision with tailored reconstruction as an effective diagnostic and therapeutic strategy in selected cases of localized PCFCL involving the nasal region.

## Introduction

Primary cutaneous lymphomas (PCLs) are a heterogeneous group of extranodal non-Hodgkin lymphomas characterized by exclusive skin involvement at the time of diagnosis, without evidence of systemic disease. Although uncommon, PCLs represent a clinically relevant entity, with an estimated global incidence of approximately 0.3 cases per 100,000 persons per year. Based on immunophenotype, they are broadly classified into T-cell and B-cell lymphomas, with primary cutaneous B-cell lymphomas (PCBCLs) accounting for nearly 20%-25% of all PCLs [1].

Among PCBCLs, primary cutaneous follicle center lymphoma (PCFCL) is the most frequent subtype, representing approximately 50%-60% of PCBCLs. PCFCL typically affects middle-aged and elderly patients and most commonly involves the head and neck region or the trunk. From a biological standpoint, it is considered an indolent lymphoma with a favorable clinical course and reported five-year disease-specific survival rates exceeding 95% when appropriately managed [2].

Despite its indolent behavior, the clinical presentation of PCFCL can be highly variable. Lesions often manifest as solitary or grouped erythematous papules, plaques, or nodules, which may resemble benign inflammatory dermatoses or non-melanoma skin cancers. This overlap frequently leads to diagnostic delays, particularly when initial biopsies are limited in size or not representative of the full lesion. Such challenges are magnified when PCFCL arises in cosmetically and functionally sensitive areas, such as the nasal dorsum and tip, where both diagnostic accuracy and therapeutic planning are critical [3]. Histopathologically, PCFCL is characterized by a dermal lymphoid infiltrate with a follicular or follicular-diffuse architecture, composed predominantly of centrocytes and centroblasts, features that may be missed in small or superficial biopsies [1,3].

Management of localized PCFCL generally relies on skin-directed therapies, including surgical excision or radiotherapy. Surgical treatment plays a particularly important role in cases involving solitary lesions or anatomically complex regions, as complete excision allows for definitive histopathological diagnosis, margin assessment, and effective local disease control [4]. However, oncologic surgery of the nasal region poses unique reconstructive challenges, requiring careful consideration to preserve nasal contour, airway function, and aesthetic subunits. In this context, the involvement of the plastic surgeon is essential to achieve optimal oncologic and reconstructive outcomes [5].

We present the case of an elderly male patient with PCFCL involving the nasal dorsum and tip, initially misdiagnosed and managed as a benign inflammatory condition, who ultimately underwent wide surgical excision with immediate reconstruction using a full-thickness skin graft harvested from an upper blepharoplasty. This case underscores the clinical relevance of maintaining a high index of suspicion for persistent inflammatory facial lesions, as well as the importance of adequate tissue sampling, clinicopathologic correlation, and individualized surgical planning in anatomically sensitive regions. This article has been prepared in accordance with the Surgical CAse REport (SCARE) 2025 guidelines [6].

## Case presentation

A 79-year-old male patient was referred for surgical evaluation due to a persistent cutaneous lesion involving the nasal dorsum and nasal tip. The lesion was associated with intense pruritus and persistent erythema, presenting as an ill-defined, infiltrated erythematous plaque that significantly affected the patient’s quality of life. At the time of surgical referral, the patient reported a symptom duration of approximately four months.

Prior to surgical referral, the patient was evaluated by a dermatologist. Given the clinical suspicion of basal cell carcinoma, a small incisional skin biopsy was performed; however, histopathological analysis was negative for basal cell carcinoma, and no definitive diagnosis was established. The patient was subsequently managed with topical treatments, and the erythema was attributed to repetitive scratching. Despite this approach, the lesion persisted, and the pruritus remained severe, prompting further evaluation.

As part of the preoperative assessment, systemic staging was performed, including a chest radiograph and a positron emission tomography (PET) scan, both of which demonstrated no evidence of metastatic disease or nodal involvement. These imaging studies were not available for later review, as the patient subsequently relocated to the United States and retained the original records.

Given the persistence of symptoms, the absence of a definitive diagnosis, and the anatomical location of the lesion, surgical excision with oncologic intent was planned. The procedure was performed on December 12, 2024, in a private surgical setting. A wide excision of the erythematous nasal skin involving the dorsum and nasal tip was carried out.

**Figure 1:**
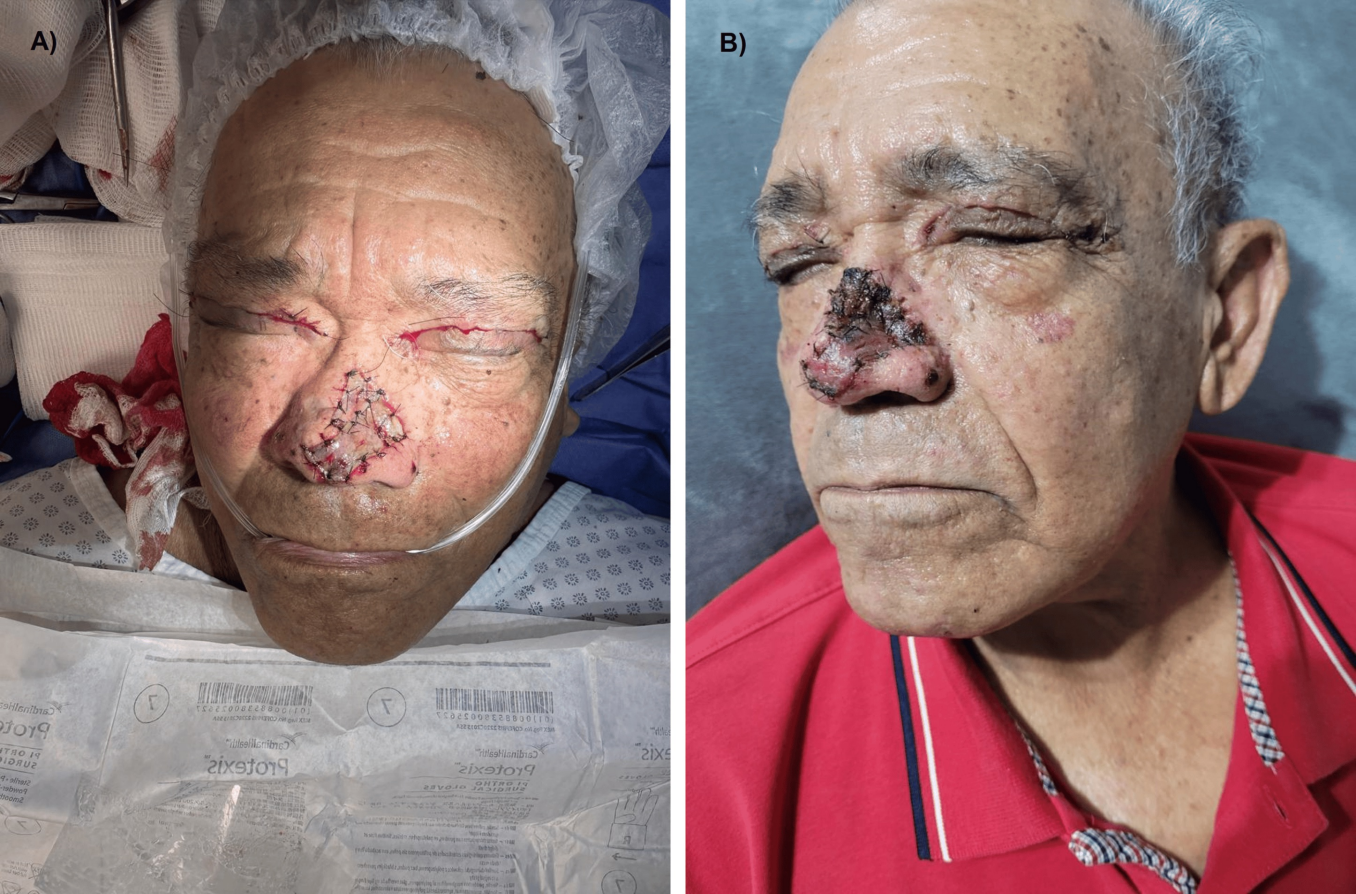
Clinical appearance following surgical excision and early postoperative evolution. (A) Immediate postoperative view after wide excisional biopsy with oncologic intent of the nasal dorsum and tip, with the surgical defect reconstructed using a full-thickness skin graft harvested from an upper blepharoplasty and secured in situ. (B) Early postoperative appearance five days after surgery, showing initial graft integration and resolution of immediate postoperative changes. Written informed consent was obtained from the patient for the publication of this image.

In the same operative session, a bilateral upper blepharoplasty was performed. The excised upper eyelid skin was utilized as a full-thickness skin graft for nasal reconstruction, allowing immediate coverage of the surgical defect while preserving nasal contour and function. The graft was appropriately contoured and secured to the recipient site. The immediate postoperative course was uneventful, with no early surgical complications (Figures 1A, 1B).

To facilitate a clear understanding of the diagnostic process, surgical management, and postoperative evolution, the key clinical events of this case are summarized in a chronological timeline (Table 1), highlighting the sequence from the initial presentation to long-term follow-up.

**Table 1:** Clinical timeline summarizing the diagnostic evaluation, surgical management, and postoperative course of the patient.

Relative time	Clinical event
Approximately four months prior to surgery	A 79-year-old male patient developed a persistent cutaneous lesion involving the nasal dorsum and nasal tip, associated with intense pruritus and persistent erythema. The symptom duration was reported by the patient at the time of surgical referral.
Preoperative period	Initial evaluation by dermatology. A small incisional skin biopsy was performed due to clinical suspicion of basal cell carcinoma; histopathological analysis was negative for basal cell carcinoma, and no definitive diagnosis was established.
Preoperative period	The patient received topical treatments, and the erythema was attributed to repetitive scratching. Despite conservative management, the lesion and severe pruritus persisted.
Preoperative staging	As part of the systemic evaluation, a chest radiograph and a positron emission tomography (PET) scan were performed, both showing no evidence of metastatic disease or nodal involvement. These imaging studies were not available for subsequent review, as the patient later relocated to the United States and retained the original records.
Day 0 (surgery)	Wide excisional biopsy with oncologic intent of the erythematous cutaneous lesion involving the nasal dorsum and nasal tip was performed.
Day 0 (reconstruction)	In the same operative session, an upper blepharoplasty was performed, and the excised upper eyelid skin was used as a full-thickness skin graft for nasal reconstruction.
Day 0 (immediate postoperative period)	The immediate postoperative course was uneventful, with no early surgical complications documented.
Days after surgery	Histopathological examination confirmed primary cutaneous follicle center lymphoma. Surgical margins were free of tumor involvement, with a closest margin of 1 mm in the smaller specimen. Additional findings included dermal interstitial fibrosis and chronic sun-related changes.
Early postoperative period	The patient showed favorable clinical evolution with appropriate graft integration, adequate wound healing, and no evidence of infection, dehiscence, skin necrosis, or nasal functional impairment.
12-month follow-up	Clinical evaluation and photographic documentation demonstrated a mature, well-integrated scar, preservation of nasal contour, and no clinical evidence of local recurrence, with satisfactory functional and aesthetic outcomes.

**Figure 2:**
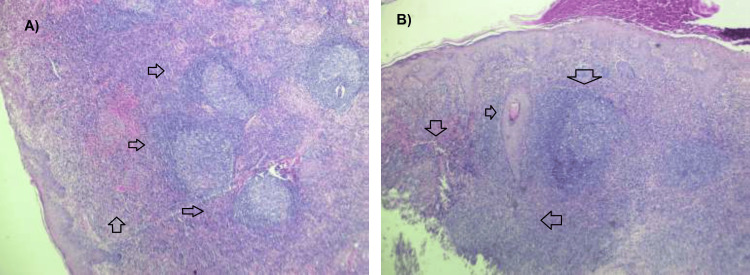
Histopathological features of primary cutaneous follicle center lymphoma. (A) Low-power histopathological view showing a dense dermal lymphoid infiltrate arranged in a predominantly follicular pattern, with preservation of follicle-like structures and involvement of both superficial and deep dermis (arrows) (hematoxylin and eosin stain). (B) Low-power section demonstrating dermal infiltration by atypical lymphoid cells with follicular architecture (arrow) beneath an intact epidermis, consistent with primary cutaneous follicle center lymphoma (hematoxylin and eosin stain).

Histopathological examination of the excised nasal specimens revealed dermal infiltration by atypical mature lymphoid cells arranged predominantly in follicular patterns with germinal centers, involving both superficial and deep dermis. Moderate mitotic activity was observed. These findings were consistent with PCFCL. Surgical margins were free of tumor involvement; however, in the smaller specimen, the lesion was located 1 mm from the closest resection margin. Additional findings included dermal interstitial fibrosis and chronic sun-related changes with dermal elastosis (Figures 2A, 2B).

**Figure 3:**
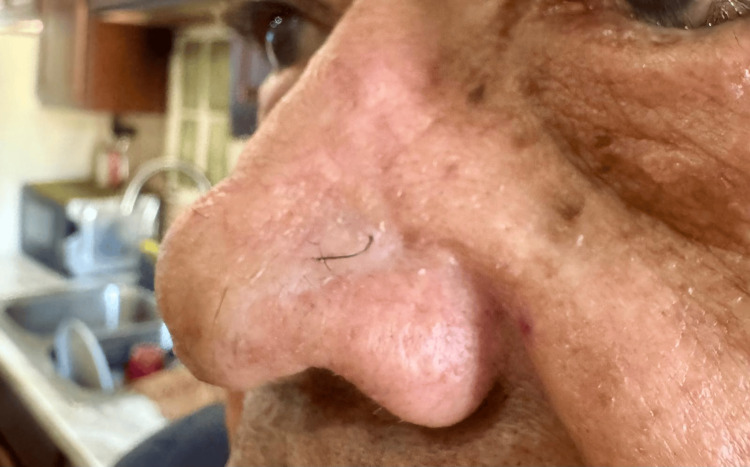
Late postoperative follow-up showing a well-healed nasal reconstruction with satisfactory contour, appropriate skin graft integration, and no clinical evidence of local recurrence at one year after surgery.

The early postoperative period was characterized by satisfactory wound healing, with appropriate graft integration and no evidence of infection, dehiscence, skin necrosis, or nasal functional impairment. At 12 months of follow-up, clinical evaluation and photographic documentation demonstrated a mature, well-integrated scar, preservation of nasal contour, and no clinical evidence of local recurrence (Figure 3).

Both functional and aesthetic outcomes were considered satisfactory. 

## Discussion

PCFCL is an indolent subtype of PCBCL that generally carries an excellent prognosis when appropriately diagnosed and treated. However, despite its favorable biological behavior, PCFCL may pose significant diagnostic challenges, particularly when presenting with atypical clinical features or in anatomically sensitive regions. The present case illustrates several of these challenges, including misleading inflammatory symptoms, an initial non-diagnostic biopsy, and involvement of the nasal dorsum and tip, an uncommon and surgically demanding location.

Delayed or incorrect initial diagnosis is a recurring theme in the literature on cutaneous B-cell lymphomas. Huang and Liu described a case of primary cutaneous diffuse large B-cell lymphoma, leg type, that clinically mimicked cellulitis, resulting in delayed recognition of the underlying malignancy [7]. Although the histologic subtype in their report was biologically aggressive and distinct from PCFCL, the clinical overlap is relevant. In our patient, persistent erythema and intense pruritus led to an initial interpretation of an inflammatory or reactive process, and symptoms were attributed to repetitive scratching. This highlights how nonspecific inflammatory manifestations can obscure the diagnosis of cutaneous lymphomas, regardless of their eventual aggressiveness.

The limitations of small or superficial biopsies in establishing a definitive diagnosis of PCFCL have also been emphasized by Choi et al., who reported unusual clinical behavior following skin biopsy in a case of PCFCL [4]. As discussed by these authors, limited tissue sampling may fail to capture the characteristic follicular architecture required for diagnosis, particularly when lesions are heterogeneous or predominantly dermal. This observation closely parallels our case, in which an initial incisional biopsy aimed at ruling out basal cell carcinoma was non-diagnostic, delaying appropriate management until a more extensive surgical excision was performed.

Anatomical location further compounds these diagnostic and therapeutic challenges. While PCFCL commonly affects the head and neck region, involvement of the nasal dorsum and tip is relatively uncommon and carries unique implications. Wei et al. reported a facial case of diffuse large B-cell lymphoma treated surgically, emphasizing that facial lymphomas, even when biologically aggressive, may be amenable to surgical management in carefully selected cases [8]. Although their case involved a different histologic subtype, their discussion supports the concept that surgery can play a meaningful role in both diagnosis and local disease control in facial lymphomas, particularly when systemic disease is excluded.

Similarly, Abeles et al. described an unusual case of diffuse large B-cell lymphoma involving the nasal bone and palate, underscoring the rarity of lymphomatous involvement in nasal structures and the importance of maintaining a broad differential diagnosis for nasal lesions [9]. Their report reinforces the notion that lymphomas of the nasal region (whether cutaneous, soft tissue, or osseous) may present atypically and require a high index of suspicion, as well as close collaboration between surgical and pathology teams.

From a therapeutic perspective, management of localized PCFCL traditionally includes skin-directed approaches, most commonly surgical excision or radiotherapy. Surgical excision offers several advantages in appropriately selected patients: it provides sufficient tissue for definitive histopathological diagnosis, allows direct assessment of surgical margins, and may achieve durable local disease control. In anatomically complex regions such as the nasal dorsum and tip, however, there is no universal consensus regarding optimal surgical margin width, and margin selection must be individualized based on tumor behavior, anatomical constraints, and reconstructive considerations. In the present case, wide excision with oncologic intent achieved histologically negative margins, albeit with close proximity in one specimen, a finding that has been reported as acceptable in selected facial locations when balanced against functional and aesthetic preservation and followed by close clinical surveillance [10].

Radiotherapy represents a well-established alternative or adjuvant modality for localized PCFCL, particularly in patients who are poor surgical candidates, have unresectable disease, or present with residual or recurrent lesions. Nevertheless, in facial regions such as the nose, radiotherapy may be associated with cosmetic and functional sequelae, and its routine use as first-line therapy must be carefully weighed against surgical options. In this patient, primary surgical management was favored given the localized nature of the lesion, the feasibility of complete excision, and the opportunity for immediate reconstruction [10].

Reconstruction of nasal defects following oncologic excision adds an additional layer of complexity. The use of a full-thickness skin graft harvested from an upper blepharoplasty, as performed in this case, allowed immediate coverage of the surgical defect while preserving nasal contour and respecting aesthetic subunits. This approach highlights the critical role of the plastic surgeon in integrating oncologic safety with functional and aesthetic outcomes, particularly in indolent facial lymphomas, where quality-of-life considerations are central.

Finally, the favorable outcome observed in this patient is consistent with the indolent biological behavior of PCFCL reported in the literature. Unlike aggressive cutaneous B-cell lymphomas, PCFCL is associated with excellent long-term survival and low rates of systemic progression when appropriately staged and treated. The absence of systemic disease on preoperative imaging and the lack of local recurrence at 12-month follow-up further support the effectiveness of a surgical, skin-directed approach in carefully selected patients.

Overall, this case highlights the potential for PCFCL to masquerade as a benign inflammatory disease, the diagnostic limitations of small biopsies, and the unique challenges posed by nasal involvement. While the conclusions drawn from a single case are inherently limited and cannot be generalized, this report provides clinically relevant insights into diagnostic reasoning, surgical decision-making, and reconstructive strategies in an uncommon facial presentation. It also demonstrates that wide surgical excision combined with thoughtful reconstruction can achieve both diagnostic clarity and durable local control, reinforcing the importance of multidisciplinary collaboration and individualized surgical planning in the management of facial cutaneous lymphomas.

## Conclusions

PCFCL may present with nonspecific inflammatory features that mimic benign dermatologic conditions, particularly when involving anatomically sensitive regions such as the nasal dorsum and tip. This case emphasizes the importance of maintaining a high index of suspicion in persistent pruritic and erythematous facial lesions and highlights the limitations of small, non-representative biopsies. Wide surgical excision provided definitive diagnosis and effective local disease control in this patient, while immediate reconstruction with a full-thickness skin graft harvested from an upper blepharoplasty allowed preservation of nasal contour and satisfactory functional and aesthetic outcomes. In selected cases of localized PCFCL, surgical management performed by experienced reconstructive surgeons can play a central role in both diagnosis and treatment, underscoring the value of individualized, multidisciplinary care.
